# Partner Experiences of “Near-Miss” Events in Pregnancy and Childbirth in the UK: A Qualitative Study

**DOI:** 10.1371/journal.pone.0091735

**Published:** 2014-04-09

**Authors:** Lisa Hinton, Louise Locock, Marian Knight

**Affiliations:** 1 Health Experiences Research Group, Nuffield Department of Primary Health Care Sciences, University of Oxford, Oxford, United Kingdom; 2 National Perinatal Epidemiology Unit, University of Oxford, Oxford, United Kingdom; University of Stirling, United Kingdom

## Abstract

**Objective:**

Severe life-threatening complications in pregnancy that require urgent medical intervention are commonly known as “near-miss” events. Although these complications are rare (1 in 100 births), there are potentially 8,000 women and their families in the UK each year who live through a life-threatening emergency and its aftermath. Near-miss obstetric emergencies can be traumatic and frightening for women, and their impact can last for years. There is little research that has explored how these events impact on partners. The objective of this interview study was to explore the impact of a near-miss obstetric emergency, focusing particularly on partners.

**Design:**

Qualitative study based on narrative interviews, video and audio recorded and transcribed for analysis. A qualitative interpretative approach was taken, combining thematic analysis with constant comparison. The analysis presented here focuses on the experiences of partners.

**Participants:**

Maximum variation sample included 35 women, 10 male partners, and one lesbian partner who had experienced a life-threatening obstetric emergency.

**Setting:**

Interviews were conducted in participants’ own homes.

**Results:**

In the hospital, partner experiences were characterized by powerlessness and exclusion. Partners often found witnessing the emergency shocking and distressing. Support (from family or staff) was very important, and clear, honest communication from medical staff highly valued. The long-term emotional effects for some were profound; some experienced depression, flashbacks and post-traumatic stress disorder months and years after the emergency. These, in turn, affected the whole family. Little support was felt to be available, nor acknowledgement of their ongoing distress.

**Conclusion:**

Partners, as well as women giving birth, can be shocked to experience a life-threatening illness in childbirth. While medical staff may view a near-miss as a positive outcome for a woman and her baby, there can be long-term mental health consequences that can have profound impacts on the individual, but also their families.

## Introduction

Severe life-threatening obstetric complications, are commonly known as “near-miss” events. Women experiencing these complications require urgent life-saving medical intervention [1]–[3]. The causes of these “near-miss” events are varied but include pre-eclampsia, haemorrhage, thrombosis and sepsis and may in some cases require an emergency hysterectomy or preterm delivery. Estimates suggest that for every 100 women giving birth in England, one will have a near-miss event, and that for every maternal death there are 100 near-misses [4]. Potentially 8,000 women and their families in the UK each year have to cope with a life-threatening emergency and its aftermath. Some women experience birth trauma that can have long lasting consequences [5]–[8]. Recent studies of near-misses from around the world [9]–[12]have drawn attention to the potential for long-term psychological and emotional impact of maternal morbidities. Women experience fear, frustration, disempowerment and shock during the immediate emergency, and symptoms of anxiety, alienation and flashbacks in the aftermath.

However, there is very little work that explores the fathers’ or partners’ experiences of traumatic birth associated with near-miss events. In the UK 98% of male partners attend the birth of their babies [13], [14] however they may still feel excluded at certain points, even though physically present [15], [16]. While most find the moment of birth exciting and wonderful, many feel helpless and distressed to see their partners in pain during labour. They often fail to live up to their own expectations, and are confused about their role[16]–[21]. The sparse literature on partner’s fears during childbirth found fear of their partner dying in childbirth was common [22], [23]. The risk of depression or PTSD in partners after traumatic childbirth is under-researched [24], [25].

There is very little evidence on the effects on partners of witnessing severe pregnancy complications. Snowdon et al. in a study of severe postpartum haemorrhage found male partners appreciated that they were only witnesses in an emergency but felt forgotten and undermined by the lack of communication [9]. They were rendered passive observers, powerless to help. Their findings echo work on male partners experiences of other aspects of pregnancy and childbirth [26], [27].

The aim of this study was to explore the impact of a near-miss obstetric emergency, focusing particularly on partners, drawing on qualitative interviews with both women and partners who experienced a near-miss event in childbirth in the UK as part of the National Maternal Near-miss surveillance programme (UKNeS).

## Methods

### Ethics Statement

Ethics committee approval was given for this study by the Berkshire Ethics Committee, 09/H0505/66. All participants gave informed consent before taking part and have given written consent to their interview data being included in publications.

In 2010–2012, with ethics approval, we invited women to take part in a study of experiences of near-miss maternal morbidity (life-threatening complication in pregnancy and childbirth). We also asked the women’s partners (fathers and one lesbian partner) to take part.

### The Sample

We aimed for a maximum variation sample [28], [29] of women living in the United Kingdom who had experienced a near-miss event in childbirth, defined as “severe maternal illnesses which, without urgent medical attention, would lead to a mother’s death” [1]. We aimed to cover a wide range of conditions in our sample, based on the five principal causes of direct maternal deaths identified in the three most recent maternal death enquiry reports; thrombosis and thromboembolism, hypertensive disorders of pregnancy, haemorrhage, amniotic fluid embolism, and sepsis (see Table 1) [30]. Our sample included 35 women, 10 male and one lesbian partner (see Table 2). We sought to interview those who had experienced their near-miss recently (e.g. 14 weeks) and those who were reflecting back on near-misses experienced several years previously.

**Table 1:** Conditions experienced by women interviewed.

Condition*	
Uterine rupture	4 women (2 partners)
Haemorrhage	5 women (2 partners)
Haemorrhage and hysterectomy	9 women (2 partners)
Placenta praevia	3 women (1 partner)
Placenta percreta	2 women
Placental abruption	1 woman (1 partner)
Amniotic Fluid Embolism	3 women (2 partners)
Pulmonary Embolism	5 women (1 partner)
Pre-eclampsia	2 women
HELLP syndrome	2 women (1 partner)
Septicaemia	2 women
Other (e.g. appendicitis, failed intubation)	4 women (1 partner)

*some women had multiple morbidities, so they appear in more than one category.

**Table 2:** Socio-demographic characteristics of 46 participants.

Characteristic	No of participants
**Age at the time of interview (years)**	
21–30	3
31–40	30
40+	13
**Age at time of near miss event (years)**	
21–30	11
31–40	30
40+	5
**Sex**	
Women/mothers	35
Fathers/Partners	11 (10 men, one lesbian partner)
**Occupation**	
Professional	20
Other non-manual	13
Skilled manual	4
Unskilled manual	2
Other (such as housewife or student)	7
**Ethnic Group**	
White British	42
British Pakistani	1
White Australian	2
White Israeli	1
**Time since near miss**	
<1 year	9
1–2 years	16
2–5 years	15
5–9 years	4
10+ years	2

Recruitment packs were distributed through a number of routes to ensure a wide, varied sample. Routes included support groups, the National Childbirth Trust, social network forums (Mumsnet and Netmums), newspaper advertisement, intensive care clinicians contacted through the Intensive Care National Audit and Research Centre (ICNARC), an advertisement in the UK Obstetric Surveillance System (UKOSS) newsletter, and word of mouth. To try and reach a wider ethnic minority population, we had the recruitment packs translated into Bengali and distributed through a consultant in an east London hospital.

We did not interview all those who volunteered, but sought to ensure that we included a representative range of conditions and times since the event, as we were keen to understand the longer-term effects of a near-miss event. Our sample included broad socio-economic diversity.

One of the authors (LH) interviewed participants in the setting of their choice (usually their home) for between one and three hours. Having signed a consent form, participants were asked about their or their partner’s experiences of pregnancy and life-threatening illness. The interview started with an open ended narrative section where respondents described what had happened. When the narrative was finished, a semi-structured interview guide with prompts was used to explore any relevant issues that had not already emerged, including their recovery and family life since their near-miss. The interviews were all audio or videotaped and transcribed verbatim. The option of audio or video interviews was offered to respondents to ensure compatibility with disseminating the findings from the study on the Healthtalkonline website (http://healthtalkonline.org/peoples-experiences/pregnancy-children/conditions-threaten-womens-lives-childbirth-pregnancy). Analysis was undertaken using the verbatim transcripts, not the audio or video data to allow for a single analytical technique irrespective of recording method. The transcripts were checked and then returned to the participants so they could read and modify the text if necessary. At this stage participants were asked to sign a copyright form giving us permission to use the content of their interviews on our website and in publications, research, education, lectures and broadcasting. They also signed another form indicating the name they wished us to use on the website. Several chose a pseudonym.

### Analysis

The data was read and re-read, a coding frame was constructed and the data coded. Emergent themes were then examined across the whole data set as well as in the context of each person’s interview. A qualitative interpretive approach was taken, combining thematic analysis with constant comparison [31], [32]. NVIVO 9 was used to facilitate the analysis [33].

The analysis presented here focused on the experiences and support needs of partners. We have included data from interviews with women, as well as their partners. Memories of near-miss events are often partial (for both the mother and her partner) because the women may be unconscious for periods of time, and their partners may not have been with them during life-saving procedures. By including data from both, we attempt to construct as complete a picture of these events as possible. Including women’s indirect accounts also gives an intimate perspective on what their partners have been through, which they might not have been willing to express themselves in an interview.

## Findings

We interviewed people who had recently experienced a near-miss, and those who were looking back on experiences that took place several years previously. In presenting our findings we indicate how long after the event respondents were interviewed. While for some, their experiences did not appear to have had long lasting effects, for others the aftershocks were still rippling through the family, years later. Recall appeared to be as vivid after several years as it was after several months.

### 1. Experiences in Hospital

Because of the wide range of illnesses that can result in a near-miss, emergency experiences varied. While for some there was a long build up during pregnancy or labour before things started to go wrong (e.g. where the woman had a diagnosis of placenta praevia), for others the emergency developed very quickly.

#### Powerlessness & exclusion

Partner experiences in hospital were often characterized by powerlessness and exclusion. Mark and his wife were rushed to hospital in an ambulance when she started bleeding. He described how he tried to remain calm. Once they arrived at the hospital, Mark felt all he could do was be passive.

*All hell broke loose [….] I just watched what was going on. I had nothing to do. I was powerless, completely powerless.”* (Mark, interviewed 4 years later)

Amy and Sally, lesbian partners, were both in the delivery suite when Amy started to haemorrhage. Sally was asked to leave the room. She was left alone while the emergency was being managed, and was not given an explanation as to why she needed to leave.

*I didn’t have a clue what was going on. I didn’t know. You know, all I knew was suddenly the room was full of people. You know, absolutely rightly no one was talking to me about what was going on apart from the anaesthetist and so they said, “Perhaps you should leave.”*

*[um] And of course, I think whilst they say that that’s probably the right thing for me to do, but of course I was starting to think, well why are you asking me to leave? What the hell is going on?* (Sally, interviewed one year later)

John’s partner haemorrhaged several times before doctors were forced to perform a hysterectomy to save her life. He was left for hours with no news about his wife’s condition.

*They said, “Oh God, she’s bleeding again, she’s bled again.” So they took her to the other side of the room literally, to where the operating theatre was, and then that’s when I could hear [partner] saying, “Look, get him out, get him out.” I thought oh God, what’s going on? So they pushed me out the door and obviously they didn’t have time, chance to say anything. So I was sort of left wandering around the hospital for another two or three hours.* (John, interviewed one year later)

#### Witnessing

Once the very immediate emergency was over, partners were often able to visit the woman’s bedside. She was often being cared for in an intensive care unit. Viewing their critically ill partner was for some a profound shock and very distressing. Sarah was taken to intensive care after she haemorrhaged. She described how shocking her husband, Rob, found the experience of seeing his wife unconscious.

*He found it very hard to cope with the fact that the nurses brought him in and he had to say goodbye before they transferred me, when I was sort of in this coma. And it was a complete shock to him, he had no idea that this could happen… he was so unprepared.* (Sarah, interviewed 5 years later)

Dean’s wife was in intensive care after she developed amniotic fluid embolism. At first he found it too distressing to look at her.

*She was like on the bed and it looked like a hundred doctors around her and she was swollen…and I just took one look and I just come back out because I couldn’t face seeing her like that.* (Dean, interviewed 2 ½ years later)

Craig’s wife was taken to intensive care for a few hours after her emergency caesarean. When he was taken in to the unit to see her he misunderstood the situation and thought she was dead.

*And all going through my mind was how am I going to cope without [wife]? Because I actually thought she was dead. And they were, you know, just holding her on a life support machine until such time. Because nobody turned round and said, “Oh she’ll be okay shortly or…” And you know, and [um] [2 sec pause] I just stood there, probably for about an hour. Just stroking her hands.* (Craig, interviewed 8 months later)

#### Contact with their baby

Another aspect of the emergency that partners found difficult was dealing with their newborn baby. For some there was the shock of discovering that their baby was ill and needed to be in neonatal intensive care. But for others, there was the challenge of looking after their newborn while the baby’s mother was seriously ill.

Dean’s wife was in intensive care and his daughter in neonatal unit. He split his time between them.

*So I was basically spending two or three hours with [daughter], back up at the hospital, back up the corridor to Intensive Care and just back and forth.* (Dean, interviewed 2 ½ years later)

Partners we talked to described how hard it was being pulled in two directions – joy at the arrival of their new baby and terrible fear for the health of the mother. James’s partner had amniotic fluid embolism after their first daughter was born, and was in intensive care.

*I was very aware that kind of my head was being pulled in two different directions at the same time. Because there was this kind of, I’d got this daughter, I’d also got this unconscious partner.* (James, interviewed 3 years later)

James was sent home with his newborn daughter while his partner remained in hospital. It was a steep learning curve.

*It was a really sudden thing because [er] they suddenly decided for [partner] to go into this isolation ward and they said, I was like, “Well what happens with [daughter]?” And they said, “Well she’ll go back down to the child thing.” And then suddenly they kind of said, [um] “No she can’t go in there.” […[they said, “You’re leaving the hospital.”* (James, interviewed 3 years later)

#### Support

Several participants described valuable/helpful support during the emergency from either relatives or medical staff. Simon was glad he had his mother there with him, Sarah made sure her father was with her husband during her operation, and Alison described how staff made sure her husband had someone with him. Small acts of support from medical staff made a big impact. Mike and Joanna’s baby was stillborn after she had an internal haemorrhage, and he refused to leave her side as she went through the for surgery to deliver the baby. The anaesthetist put her arm around him as he held his stillborn baby – a simple act he found very comforting.

*I obviously thought I’d be coming out of there and not only having to explain to my daughter that she hasn’t got a sister, but you know, she hasn’t got a mummy as well. And the realisation of it was just immense really. [um] But as I say, the one person who took an interest there was that consultant anaesthetist. [um] You know, I just remember this one kind of scene really. This one moment where I had my arm round [wife]. Obviously [wife] was out for the count, and I was holding my daughter, and I was just, you know, a mess basically and it was the anaesthetist who actually put her arm round me and she was stroking [wife’s] hair as well.* (Mike, interviewed one year later)

### Communication

How well staff communicated during or after the emergency was very important. Michael, whose wife developed HELLP syndrome, a rare liver and blood clotting disorder (the letters stand for each part of the condition, ‘H’ haemolysis, ‘EL’ elevated platlets, ‘LP’ low platelet count). He felt the communication from doctors during the emergency was very good, if overwhelming.

*I think they did a wonderful job of trying to explain it in a way that a medical dummy like me could sort of understand things.* (Michael, interviewed 11 weeks later)

But other partners we talked to did not feel the communication was good at all. John felt he was not listened to during the early stages of his partner’s emergency, “a second class citizen”, and was left for three or four hours in the blood stained delivery suite before anyone came back to give him news. When Hannah developed complications after the birth of their daughter, her husband, Simon, was left waiting without any news. He twice walked up to the surgery doors and was told to go away.

*I think that was just a horrible experience for him. He didn’t know anything, he thought I had died.* (Hannah, interviewed 2 ½ years later)

While people appreciated it is difficult for staff to communicate when dealing with the emergency of saving the woman’s life, when they did communicate with the partner it made a welcome difference.

*I remember it being kind of you know, very vague medical speak, and I actually had to say at the end, “So there’s a chance she won’t make it?” I think I just wanted a meat and potatoes kind of conversation. I didn’t want some fancy words.* (James, interviewed 3 years later)

### 2. Father’s/Partner’s Emotional Recovery

Some partners told us it took time to recover from witnessing their partner’s life-threatening emergency. Craig’s wife was in intensive care after delivering their twins. He was interviewed eight months later and said the experience was hard to put behind them.

*I’d like to believe that we’ve put it behind us, but we still talk about it, so we haven’t quite put it behind us. It still manifests itself sometimes.* (Craig, interviewed 8 months later)

Mark’s wife was rushed to hospital in an ambulance and had an emergency caesarean after a placental abruption. He has not felt traumatised by what he witnessed, but feels doctors could have taken a few minutes to explain to him what had happened and made sure that he was coping.

*Afterwards I thought, there was space there, to actually involve me a bit more in what was going on, and it wouldn’t have taken too much effort, given that they were all ready and able to dash in and you know, eight or ten of them there, at the crash, to keep one of them behind for a few minutes, just to make sure that I wasn’t less sturdy than I was. Because I’m a pretty sturdy guy, I think, I like to think I am. So I could withstand it, but someone who was quite as robust as me, might have really gone to pieces at that point, not knowing what was going on.* (Mark, interviewed 4 years later)

Although the partners we spoke to were all deeply affected by their partner’s life threatening experiences, for some it has had a profound impact on their long-term health, including experiencing depression, flashbacks, a breakdown or post traumatic stress disorder (PTSD) in the months/years since the emergency. Craig described the experience of seeing his wife in intensive care after the birth of their twins.

*The most stress I’ve ever been under.* (Craig, interviewed 8 months later)

He had a vasectomy to make sure they do not have to go through childbirth again.

Dean’s wife had amniotic fluid embolism and was critically ill in intensive care. He tries to hide his feelings but two and a half years on still has flashbacks. Tom’s wife had a pulmonary embolism and a haemorrhage during her second pregnancy. Interviewed a year and a half later, he described a nervous breakdown that he attributes to the stress of his wife’s prolonged illness. Rob’s wife had placenta praevia and a hysterectomy with her third pregnancy. He has found his experiences of his wife’s emergency have had a huge impact on his mental health in the subsequent five years. He has flashbacks and has been diagnosed with PTSD and depression.

*So I finally curled up in the corner and the kitchen and, and I’m not in the kitchen, I’m in that room [intensive care]. As I say I didn’t believe it when soldiers come back saying … And there am I in my kitchen, although I’m not. I’m… and it’s the strangest feeling to actually be stood somewhere but be somewhere else. It was just horrific, and my whole life has fell apart.* (Rob, interviewed 5 years later)

Some partners who had looked for support found it hard to get acknowledgement for their distress and help for their depression or flashbacks. When Rob finally plucked up courage to go and see his GP to ask for help the response he got was devastating.

*He said to me, he looked me right in the eye, and he said to me, “Mr [name],” he says. [er] “Your wife is the one that went through all the trauma, and everything else. You just need to pull yourself together and be there for your wife.” [3 sec pause] And that was it. That for me, I fell into a pit of despair from there. Because of course what am I going to come away thinking, I’m thinking, he’s right, he’s right. What is the matter with me? I’m having all these flashbacks and that. I can’t go to work, what sort of a man am I? I can’t, you know, I need to pull myself together, but equally I, I couldn’t. I, I couldn’t function.* (Rob, interviewed 5 years later)

Others found dealing with their trauma very isolating, as it was hard to talk to family and friends.

*I think men are quite difficult to get support from, It was a lot of my friends, you know, empathised quite a lot, but then you expect your close friends to be able to discuss it on a, more of a personal note, but it’s surprising how many of those really close friends found it too uncomfortable.* (Mike, interviewed one year later)

## Discussion

Although clinicians are aware that childbirth can be accompanied by life threatening complications, in modern, industrialised societies childbirth is popularly regarded as safe and routine, and awareness of the dangers is low. Women and their partners are therefore shocked and surprised to experience life-saving interventions, as demonstrated by our data and other recent research [9], [10]. These events can provoke fear and anxiety and have profound long-term consequences for mothers and their partners. In hospital contexts where medical staff quite reasonably view a near-miss as a positive outcome for mother and baby, our study suggests the potential for negative outcomes for close family members, about which hospital staff may be unaware. It highlights that partners feel side-lined and fearful during the emergency, that support and communication is highly valued but often lacking, and that for some there can be long-term mental health consequences that can have profound impacts on the individual and their families.

Our study aimed to interview people who had recent experiences, and also those looking back on experiences they had several years previously. This was to explore any long term effects these life-threatening emergencies might have had and also what, if any, impact they had on subsequent family life and size. Respondents spoke of the impact their experiences had on their mental health, and future fertility. While in some cases, the narratives were of often-told stories, in other cases the narratives were the first opportunity that partners (in particular) had been offered to talk about their experiences (e.g. Dean). The narratives often gave insights into power of the trauma and its lasting effects, even if the event was several years previously. Often respondents spoke of the isolation they experienced because their experiences were so rare. Telling their stories was an opportunity to be share those experiences, and help others in future.

Our study has limitations. The data presented here is based on interviews with 11 partners and 36 mothers. As with any qualitative study aiming for a maximum variation sample, the findings are not intended to be numerically representative. By including both partners’ experiences we attempt to build as complete a picture as possible of these emergencies. The often fast-developing nature of life-threatening obstetric complications means that there were significant parts of the experience to which neither the mother nor her partner were witness. For example, the mother might not have memories of the periods of time when she was unconscious during life saving treatment. Her partner might not have been in the room for key moments. Including mother’s indirect accounts in our analysis was important because they were able to offer an intimate insight into what their partners had been through. Women may have been able to give voice to feelings that their partners have discussed with them in private but would not be willing to express in an interview, especially in the context of the difficulties some men found in finding professional acknowledgement of their emotional reactions [26].

Although some interviews were audio recorded and others video recorded, analysis of all the interviews was undertaken on the verbatim transcripts. We mainly interviewed white British people, and although there was a spread of socioeconomic background among those interviewed, there could have been additional perspectives if the study had included a broader social and ethnic diversity. While no account is static – people’s views and interpretation of their experiences are likely to change over time – we made an effort to interview individuals who were both close to the events and also those who were talking about experiences that happened several years previously.

Partners are expected to attend birth, but can feel marginalised even at uncomplicated births. Birth is life-changing and potentially traumatic and empowering for both partners [16]. Partners see themselves as more than passive supporters; they want active engagement. They struggle with balancing their own emotions and supporting the mother. They can feel vulnerable and uncertain; yet find it difficult to admit their fears. Stoicism and self-reliance do not always help [18]. Ayers et al (2006) suggest that if a mother’s psychological symptoms go unrecognized and untreated after birth trauma, depression and long-term consequences for women may result, including isolation from their circle of friends [34]. There are direct parallels in our data, where partners found it hard to find support or appropriate forums in which to discuss their feelings.

In one of the few studies that included partners’ experiences of obstetric emergencies, there are parallel themes of disempowerment and information-deprivation, involuntary separation and exclusion from partners, anxiety about their newborns and difficulties for caring for newborn while partner still in recovery [9]. Men felt forgotten. They were involved in an emergency but felt undermined by the lack of communication and the sense that their practical and emotional needs were inadequately met. Jessop and Fox [25] explored counsellors’ experiences of counselling fathers experiencing birth trauma. Fathers were often reluctant to seek help as it contradicts social expectations that birth is a female event. The data reported here adds to growing evidence that some men may experience birth trauma [24]. These results do not only have implications for supporting the mental health of partners. Fathers have a vital role in supporting women post-natally and this in turn has a longer-term impact on the health and well-being of the mother and infant[35]–[39]. Redshaw and Henderson [14] report postnatal health tends to be better for women whose partner was more involved during labour. Where fathers are more involved post-natally there are higher breastfeeding rates. International research has drawn attention to the economic burden of emergency obstetric care [40], and in a context where men have taken extra time off work, missed promotions or lost their jobs as a result of their partner’s near-miss, this study suggests there are further issues to consider in the longer term.

International and UK national policies[41]–[44] advocate and support the involvement of fathers throughout pregnancy and childbirth. While there is little research on fathers’ experiences of traumatic, life-threatening birth, there are other research areas where learning might provide useful insights into how best to support these men and their families. Research on witnessing resuscitation has led to the development of “family presence” programmes that are well-established and have provided evidence of the benefits to patients, family members and staff [45]. A study of the impact on staff of father’s presence during the resuscitation of their newborn acknowledged that a key factor in the failure to meet the emotional and support needs of fathers appeared to be that none of the professionals involved had direct responsibility to support and communicate with him [46]. Although there is guidance about supporting parents in the delivery room in the updated European and UK newborn life-support training programmes, there is no specific guidance about ways to communicate and support the father or partner. The similarity of the themes identified in our study with this work suggests a role for the development of guidance for supporting partners during and after complicated childbirth. In addition, further investigation of the potential for extension of “family presence” programmes to mitigate some of the negative effects identified in this research may be warranted.

## Conclusion

All the partners/fathers we spoke to had been deeply affected by their partner’s life threatening experiences. For some it had a profound impact on their long-term mental health. In situations where an emergency delivery might be anticipated, such as when a woman has placenta praevia, an explanation of what might happen really helped partners prepare and cope subsequently. Staff might consider that frequent updates during the emergency help partners/fathers feel less isolated and anxious.

Our study demonstrates that (often small) personal touches of support from individual staff can make a real difference to how partners cope.

While partners may remember more about events than the woman who is ill, they still appreciate repeated explanations. Partners/fathers can find seeing their partner in high dependency or intensive care very traumatic, and may need support from staff and family members to enable them to visit their partner, understand that the situation is not hopeless and their partner may recover, and to come to terms with what has happened.

Long-term mental health problems in partners/fathers after a near-miss experience may have a big impact financially, practically and emotionally and families may need additional support in this event. They often felt that counseling could have been beneficial, if it had been offered. However, clinicians should take into account that partners/fathers who experience mental health symptoms do not necessarily seek help.
