# Correction: Partner Experiences of “Near-Miss” Events in Pregnancy and Childbirth in the UK: A Qualitative Study

**DOI:** 10.1371/journal.pone.0108803

**Published:** 2014-09-17

**Authors:** The PLOS ONE Staff

The affiliation for the second author is incomplete. Louise Locock should have the following added to her affiliation: Health Experiences Fellow, NIHR Oxford Biomedical Research Centre.
